# Insulin-like growth factor-1 (IGF-1) as predictor of cardiovascular mortality in heart failure patients: data from the T.O.S.CA. registry

**DOI:** 10.1007/s11739-022-02980-4

**Published:** 2022-04-21

**Authors:** Alfredo De Giorgi, Alberto Maria Marra, Massimo Iacoviello, Vincenzo Triggiani, Giuseppe Rengo, Francesco Cacciatore, Ciro Maiello, Giuseppe Limongelli, Daniele Masarone, Francesco Perticone, Pasquale Perrone Filardi, Stefania Paolillo, Antonio Mancini, Maurizio Volterrani, Olga Vriz, Roberto Castello, Andrea Passantino, Michela Campo, Pietro Amedeo Modesti, Andrea Salzano, Roberta D’Assante, Michele Arcopinto, Valeria Raparelli, Fabio Fabbian, Angela Sciacqua, Annamaria Colao, Toru Suzuki, Eduardo Bossone, Antonio Cittadini, T.O.S.CA. Investigators, A. Cittadini, M. A. ArcopintoSalzano, L. Saccà, M. G. Monti, R. Napoli, M. Matarazzo, F. M. Stagnaro, A. Schiavo, P. Valente, E. Bossone, F. Ferrara, V. Russo, M. Malinconico, R. Citro, E. Guastalamacchia, M. Iacoviello, M. Leone, V. Triggiani, F. Cacciatore, C. Maiello, C. Amarelli, I. Mattucci, G. Limongelli, D. Masarone, P. Calabrò, R. Calabrò, A. D’Andrea, V. Maddaloni, G. Pacileo, R. Scarafile, F. Perticone, A. Belfiore, A. Sci-acqua, A. Cimellaro, P. Perrone Filardi, L. Casaretti, S. Paolillo, P. Gargiulo, A. Mancini, A. M. R. Favuzzi, C. Di Segni, C. Bruno, E. Vergani, O. Vriz, R. Castello, A. Frigo, M. Campo, M. R. Sorrentino, P. A. Modesti, D. Malandrino, R. Manfredini, A. De Giorgi, F. Fabbian, A. Puzzo, L. Ragusa, L. Caliendo, L. Carbone, A. Frigiola, T. Generali, F. Giacomazzi, C. De Vincentiis, A. Ballotta, P. Garofalo, G. Malizia, T. Suzuki, L. M. Heaney, D. Bruzzese

**Affiliations:** 1 Clinica Medica Unit, Azienda Ospedaliero-Universitaria S.Anna, Ferrara, Italy; 2 Department of Translational Medical Sciences, Federico II University, Naples, Italy; 3 Cardiology Unit, Department of Medical and Surgical Sciences, University of Foggia, Foggia, Italy; 4 Interdisciplinary Department of Medicine-Section of Internal Medicine, Geriatrics, Endocrinology and Rare Diseases, University of Bari ‘A Moro’, Bari, Italy; 5 Heart Transplantation Unit, Monaldi Hospital, Azienda Ospedaliera dei Colli, Naples, Italy; 6 Division of Cardiology, Monaldi Hospital, Azienda Ospedaliera dei Colli, University of Campania L. Vanvitelli, Caserta, Italy; 7 Department of Medical and Surgical Sciences, University Magna Græcia of Catanzaro, Catanzaro, Italy; 8 Department of Advanced Biomedical Sciences, Federico II University, Naples, Italy; 9 Operative Unit of Endocrinology, Catholic University of the Sacred Heart, Rome, Italy; 10 Department of Medical Sciences, IRCCS San Raffaele Pisana, Rome, Italy; 11 Heart Center Department, King Faisal Hospital and Research Center, Riyadh, Kingdom of Saudi Arabia; 12 Division of General Medicine, Azienda Ospedaliera Universitaria Integrata, Verona, Italy; 13 Scientific Clinical Institutes Maugeri, IRCCS, Pavia, Italy; 14 Unit of Endocrinology and Metabolic Diseases, Department of Medical and Surgical Sciences, University of Foggia, Foggia, Italy; 15 Dipartimento di Medicina Sperimentale e Clinica, Università degli Studi di Firenze, Florence, Italy; 16 Italian Clinical Outcome Research and Reporting Program (I-CORRP)-IRCCS SDN, Diagnostic and Nuclear Research Institute, Naples, Italy; 17 Department of Translational Medicine, University of Ferrara, Ferrara, Italy; 18 Clinical Medicine and Surgery Department, Federico II University, Naples, Italy; 19 Department of Cardiovascular Sciences and NIHR Leicester Biomedical Research Centre, University of Leicester, Glenfield Hospital, Leicester, UK; 20 Italian Clinical Outcome Research and Reporting Program (I-CORRP)-Cardiology Division, A. Cardarelli Hospital, Naples, Italy; 21 Italian Clinical Outcome Research and Reporting Program (I-CORRP)-Center for Pulmonary Hypertension, Thorax Clinic at Heidelberg University Hospital, Heidelberg, Germany; 22 Istituti Clinici Scientifici Maugeri SpA Società Benefit-IRCCS-Scientific Institute of Telese Terme, Telese Terme, Italy; 23 Mediterranea Cardiocentro, Naples, Italy; 24 Italian Clinical Outcome Research and Reporting Program (I-CORRP), Naples, Italy; 25 Department of Medical Sciences, University of Ferrara, Ferrara, Italy

**Keywords:** Chronic heart failure, Multiple hormonal deficiency syndrome, IGF-1 deficiency, Chronic renal failure, Heart failure with reduced ejection fraction

## Abstract

**Introduction:**

Data from the “Trattamento Ormonale nello Scompenso CArdiaco” (T.O.S.CA) registry showed that heart failure (HF) represents a complex clinical syndrome with different hormonal alterations. Renal failure represents a frequent complication in HF. We evaluated the relationship between renal function and insuline-like growth factor-1 (IGF-1) deficiency and its impact on cardiovascular mortality (CVM) in patients enrolled in the T.O.S.CA. registry.

**Methods:**

At the enrolment, all subjects underwent chemistry examinations, including circulating hormones and cardiovascular functional tests. COX regression analysis was used to evaluate factors related to CVM during the follow-up period in all populations, in high-risk patients and in the young-adult population. Also, we evaluate the effects of renal function on the CVM.

**Results:**

337 patients (41 deceased) were analyzed. CVM was related to severe renal dysfunction (HR stages IV–V = 4.86), high-risk conditions (HR 2.25), serum IGF-1 (HR 0.42), and HF etiology (HR 5.85 and HR 1.63 for valvular and ischemic etiology, respectively). In high-risk patients, CVM was related to IGF-1 levels, severe renal dysfunction and valvular etiology, whereas in young patients CMV was related to the high-risk pattern and serum IGF-1 levels.

**Conclusions:**

Our study showed the clinical and prognostic utility of the IGF-1 assay in patients with HF.

**Supplementary Information:**

The online version contains supplementary material available at 10.1007/s11739-022-02980-4.

## Introduction

Heart failure (HF) represents an important public health problem, affecting around 23 million patients worldwide [1]. The HF burden has a huge impact in economic terms, resulting in about 2% of the total health-care budget in many European countries; the overall economic cost of HF in 2012 was estimated at $ 108 billion per annum. These costs, linked to the high morbidity and health needs of an aging, rapidly expanding and industrializing population, are expected to grow progressively over time—all costs linked to the elevated morbidity, healthcare needs and mortality [2].

The HF is a complex clinical syndrome affecting different functional systems of the body. Although the causes leading to HF are mainly related to cardiac alterations, HF is undoubtedly associated with multi-organ dysfunctions affecting lungs, kidneys, brain and endocrine glands. Notably, a multihormonal deficit syndrome (i.e. alterations of thyroid, pituitary, adrenal and pancreatic hormones), characterized by changes in the catabolism/anabolism balance, has been reported in patients with HF [3, 4]. Hormonal deficiency is associated with the progressive reduction of cardiac functional capacity until cardiac cachexia occurs, thus resulting in a powerful and independent predictor of prognosis in patients with chronic HF [5]. Recently, data from the “Trattamento Ormonale nello Scompenso CArdiaco” (T.O.S.CA) registry showed that patients with HF and pre-served ejection fraction (HFpEF) had at least one endocrine dysfunction in 54% of cases, while this percentage reached 96% in the case of patients with HF and reduced ejection fraction (HFrEF) [6]. Similar results were detected in a study performed by Favuzzi et al. in 40 patients with HFpEF showing that only 2.5% of patients had no hormonal deficiencies [7]. The alteration in the synthesis of the Growth Hormone (GH) and its peripheral effector insuline-like Growth Factor-1 (IGF-1) was documented in approximately 40–60% of HF patients [8]; on the other hand, the presence of a peripheral resistance to GH appears to be associated with the development of heart failure and cardiac cachexia [9]. The GH/IGF-1 axis acts during the post-infarct ventricular remodeling that results from myocyte apoptosis; it favors vasodilation through action on nitric oxide and it improves muscle performances [10]. For these reasons, IGF-1 deficiency would seem to have important effects in terms of both HF development and worse prognosis [11]. Furthermore, among HF patients, low serum IGF-1 was associated with worse outcomes [11–13] including cardiovascular and all cause of mortality [14]. Nevertheless, other clinical studies exploring the relationships between serum IGF-1 and the prognosis of HF patients were contradictory [15, 16].

Renal failure is a well-known risk factor for further cardiovascular diseases in patients with HF [17] and, at the same time, the relationship between HF and renal function is a condition belonging to the family of the cardiorenal syndromes. Relationship between IGF-1 and renal function is still a matter of debate. In addition to heart failure, IGF-1 values seem to be associated with the risk of developing CKD, as demonstrated by Teppala et al. [18] in a population of 5388 patients. The elevated serum levels of IGF-1 were associated with the development of CKD after adjustment for different confounding factors such as age, sex, race/ethnicity, education levels, smoking, alcohol intake, body mass index, diabetes, hypertensionand serum cholesterol. On the other hand, in patients with CKD the effect of IGF-1 serum levels on mortality is opposite. In incident dialysis patients, lower serum levels of IGF-1 seem to be associated with metabolic alterations and predict the risk of mortality [19]. In arthritic rats, GH treatment decreased serum creatinine levels and IGF-1 concentrations [20].

The aim of this study was to evaluate whether a relationship between renal function and IGF-1 deficiency exist and its impact on cardiovascular mortality in patients with moderately or severely HFrEF, enrolled in the T.O.S.CA. registry.

## Methods

### Patient selection and eligibility

Data for the present analysis come from the T.O.S.CA. Registry, an out-come-oriented, multicenter, prospective observational study, designed to evaluate the prevalence of multiple hormone deficiency syndromes in patients with CHF and its impact on clinical outcomes. The study design has been already published [21]. Briefly, all patients had CHF with reduced EF (≤ 45%) and stable home therapy for at least 3 months. At the time of enrolment, all subjects underwent routine clinical chemistry examinations, such as B-type natriuretic peptides (BNP) and renal function with evaluation of serum creatinine and estimated glomerular filtration rate (eGFR) using the Chronic Kidney Disease Epidemiology Collaboration (CKD-EPI) equation [22], and cardiovascular functional assessments, such as echocardiography and evaluations the New York Heart Association (NYHA) classification, in the peripheral centers, while the evaluation of circulating hormones (thyroid hormones, GH/IGF-1 axis, total testosterone, dehydroepiandrosterone sulfate (DHEA-S), and insulin) were analyzed in a dedicated core-lab (IRCSS-SDN, Naples, Italy) collaborating with the Clinical Coordinating Center.

After enrolment, all subjects were followed as outpatients with each procedure and blood sample collection that were repeated annually during the study period. Intermediate visits were scheduled for clinical follow-up, therapy monitoring, and to collect relevant clinical event information (for mortality and hospitalization).

Clinical data and index events from enrolled patients identified by a unique identifier number were updated on a Web-based platform (URL: http://www.registrotosca.com).

Based on the analyses performed on the entire population, we searched for factors independently associated with mortality from cardiovascular events. Such analyses were performed for both the general population and two sub-classes of patients derived from it. Renal Failure was classified according to KDIGO classification [23]. We analyzed the subclass of young-adult patients (aged < 65 years) and the subclass of patients at high risk of mortality and unexpected death. The latter subgroup was defined according to the most recent guidelines about HF and is referred to patients with EF ≤ 35% and NYHA stage 2–3, who are theoretically eligible for implantable cardioverter-defibrillator placement [1]. We focused our study on the relationship between GH/IGF-1 axis, renal function, and cardiovascular mortality. The study has been conducted in accordance with Good Clinical Practice, Declaration of Helsinki 2002. The trial has been registered on Clinicaltrials.gov (NCT02335801). The study has been approved by ethical committee of “Federico II” University Hospital (n. 34/13) and all patients agreed in signing informed consent.

### Statistical analysis

Data are shown as absolute numbers, percentages, and means ± standard deviation (SD). A descriptive analysis of the whole population was performed, followed by a comparison of survivors and deceased during the follow-up period. The analysis of variables was performed by using the Chi-Squared test, the Student *t* test or the Mann–Whitney *U* test as appropriate. Moreover, in order to assess the independent factors associated with cardiovascular (CV) mortality, a COX regression analysis was performed including age, sex, chronic kidney disease stage calculated using the CKD-EPI formula [22], logarithm of BNP and IGF-1 (parameters were not normally distributed in the population), high-risk conditions. Also, we performed a Cox regression analysis to evaluate the effects of CKD-EPI stage and the estimated cardiovascular risk of patients at the time of enrollment on the mortality risk. Using Receiver operating characteristic (ROC) curve the area under the curve (AUC) was calculated for diagnostic value and accuracy of LnIGF-1 levels with the best sensitivity and specificity for given cut-off values. Hazard ratios (HRs) and their 95% Confidence Intervals (CI) were reported. SPSS 13.0 for Windows (SPSS Inc., Chicago, IL, 2004) was used for statistical analyzes.

## Results

A total of 337 patients (80.7% male sex) with a mean age of 63 ± 12 years were analyzed.

The analysis of renal function showed an average eGFR of 72.9 ± 25.4 ml/min/1.73 m^2^. Most patients (66.5%) had normal or mildly decreased renal function (KDIGO stage I and II, eGFR ≥ 60 ml/min/1.73 m^2^) [22].

After a mean follow-up period of 44 ± 11 months, the percentage of deaths was 12.2% (41 patients); the clinical and laboratory characteristics of the cohort stratify by living status are reported in Table 1. Among the 41 recorded deaths 23 (56%) patients experienced a malignant arrythmias with unsuccessful ICD intervention, 12 patients (29%) had an acute coronary syndrome and 6 patients (15%) died because of a bradyarrhythmia.

**Table 1:** Characteristics of population and relationship with the main outcome

Overall population (*n* = 337)		Survived (*n* = 296)	Deceased (*n* = 41)	*p* value
Male Sex, *n* (%)	272 (80.7)	237 (80.1)	35 (85.4)	0.53
Age (years)	63.3 ± 12.2	63 ± 12	69 ± 11	0.003
Age ≥ 65 years, *n* (%)	156 (46.3)	131 (44.3)	25 (61.0)	0.044
NYHA class [*n* (%)]				
I	38 (11.3%)	37 (12.5%)	1 (2.4%)	0.012
II	190 (56.4%)	171 (57.8%)	19 (46.3%)	
III	107 (31.8%)	87 (29.4%)	20 (48.8%)	
IV	2 (0.6%)	1 (0.3%)	1 (2.4%)	
HF etiology [*n* (%)]				
Idiopathic	142 (42.1%)	131 (44.3%)	11 (26.8%)	0.040
Ischemic	180 (53.4%)	154 (52.0%)	26 (63.4%)	
Valvular	15 (4.5%)	11 (3.7%)	4 (9.8%)	
Creatinine (mg/dl)	1.16 ± 0.63	1.11 ± 0.57	1.5 ± 0.89	< .001
eGFR _(CKD-EPI)_ (ml/min/1.72 m^2^)	72.9 ± 25.4	74.9 ± 24.3	58.8 ± 28.7	< .001
CKD-EPI stage [*n* (%)]				
I–II	224 (66.5%)	207 (69.9%)	17 (41.5%)	< .001
III	98 (29.1%)	80 (27.0%)	18 (43.9%)	
IV–V	15 (4.5%)	9 (3.0%)	6 (14.6%)	
BNP (pg/ml)	122.1 ± 205.2	108.6 ± 187.2	219.3 ± 290.2	0.001
BNP (pg/ml) (mean or median ?—IR)	43–1490	39–1490	131 1240	0.001
Ln (BNP) (pg/ml)	3.82 ± 1.39	3.73 ± 1.36	4.52 ± 1.47	0.001
Ejection fraction (%)	32.6 ± 7.2	33.3 ± 6.8	27.4 ± 8.1	< .001
IGF-1 (ng/ml)	125.4 ± 66.1	128.4 ± 67.8	103.3 ± 47.0	0.025
Ln (IGF-1) (ng/ml)	4.72 ± 0.48	4.74 ± 0.47	4.52 ± 0.51	0.005
High risk of sudden death [*n* (%)]	181 (53.7%)	150 (50.7%)	31 (75.6%)	0.003
ACEi [*n* (%)]	181 (53.7%)	164 (55.4%)	17 (41.5%)	0.34
ARBs [*n* (%)]	95 (28.3%)	87 (29.4%)	8 (19.5%)	0.31
*β*-blockers [*n* (%)]	303 (90.0%)	267 (90.0%)	36 (87.8%)	0.91
MRA [*n* (%)]	162 (48.0%)	142 (48.0%)	20 (47.8%)	0.94
ICD	95 (52.5%)	123 (41.6%)	23 (56.1)	0.28

Data are reported as number (percentage) or number ± SD (standard deviation). BNP and IGF-1 values were log-transformed before entering the comparison analyses because of their non-normal distribution. Renal function stages were reduced to three because of the small size of the sample considering patients with severe Chronic Kidney Disease (stage IV-V)

*HF* Heart Failure, *eGFR* estimated Glomerular Filtration Rate, *BNP* Brain Natriu-retic Peptide, *IGF-1* Insuline-like Growth Factor-1; *ACEi* ACE inhibitors, *ARBs* Angiotensin Receptor Blockers, *MRA* Mineralcorticoid Receptor Antagonists, *ICD* Implantable Cardioverter Defibrillator

Compared with alive patients at follow-up, deceased subjects were older (69 ± 11 vs. 63 ± 12 years, *p *= 0.003), with a more advanced NYHA class and ventricular dysfunction based on EF values (27.4 ± 8.1 vs. 33.3 ± 6.8%, *p *< 0.001), with a higher serum BNP (219.3 ± 290.2 vs. 108.6 ± 187.2 pg/ml, *p *= 0.001), with a reduced renal function (eGFR 58.8 ± 28.7 vs. 74.9 ± 24.3 ml/min, *p *< 0.001) and with lower serum IGF-1 values (103.3 ± 47.0 vs. 128.4 ± 67.8 ng/ml, *p *= 0.025). No differences were present according to treatment. HF secondary to ischemic heart disease (IHD) and valve diseases were the 2 etiologies more likely associated with cardiovascular mortality (Table 1).

COX regression analysis (Table 2) showed that the factors independently associated with the cardiovascular mortality in patients with HFrEF were as follows: (i) severe renal dysfunction (eGFR ≤ 30 ml/min/1.73 m^2^), (HR stage III = 1.63, 95% CI 0.73–3.63, *p *= 0.16; HR stages IV–V = 4.86, 95% CI 1.48–15.95, *p *= 0.009), respectively; (ii) being classified as high-risk patient of mortality/unexpected death (HR 2.25, 95% CI 1.04–4.86, *p *= 0.039), and (iii) serum IGF-1 (HR 0.42, 95% CI 0.23–0.77, *p *= 0.005). The underlying valvular etiology of cardiomyopathy was strongly associated with CV mortality (HR 5.85, 95% CI 2.02–16.94, *P *= 0.001), while the HR concerning the ischemic form was 1.63 (95% CI 0.73–3.63, *p *= 0.23).

**Table 2:** Logistic regression modelling for evaluation of the associations with the outcome in the population

	HR	95% C.I	*p* value
High-risk Patients	2.25	1.04–4.86	0.039
Ln (IGF-1)	0.42	0.23–0.77	0.005
HF etiology			
Idiopathic	*Ref.*		
Ischemic	1.63	0.73–3.63	0.23
Valvular	5.85	2.02–16.94	0.001
CKD-EPI stage			
I–II	*Ref.*		
III	1.63	0.73–3.63	0.16
IV–V	4.86	1.48–15.95	0.009

*HR* Hazard Ratio, *C.I.* Confidential Interval, *IGF-1* Insuline-like Growth Factor-1, *HF* Heart Failure

**Fig. 1:**
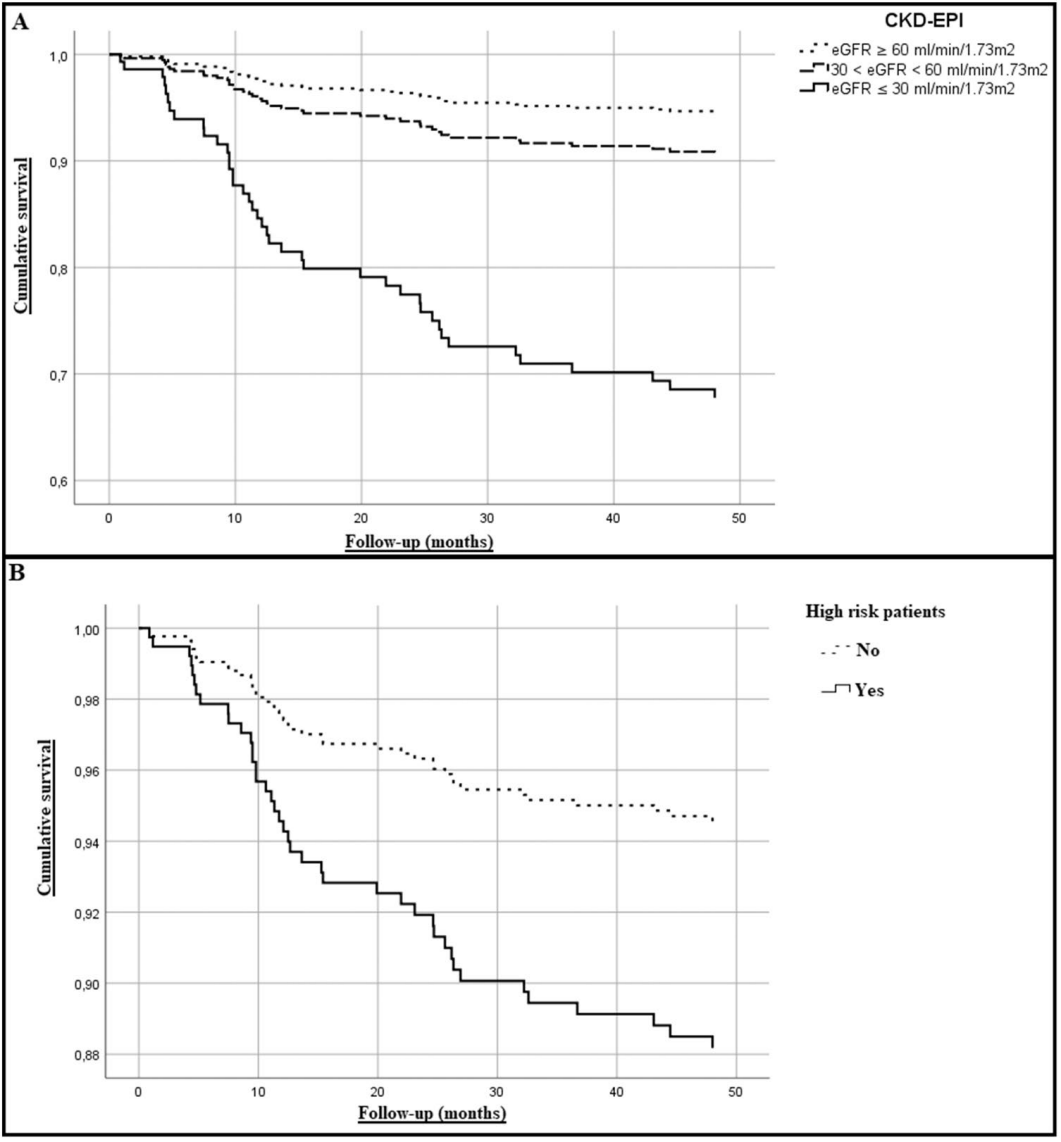
Survival functions of the population in relation with their CKD-EPI stage at the time of enrollment (tab. **A**) and with their estimated cardiovascular risk (tab. **B**)

Figure 1 shows the Kaplan Meier curves in relation to CKD-EPI stage (tab. A) and to the high-risk of death (tab. B). LnIGF-1 showed the best predictive value for incidence of CV death with the cut-off value of 4.07 ng/mL (AUC 0.608, 95% C.I. 0.51–0.70) (see Supplemental material).

In high-risk patients it was possible to highlight how the factors associated with higher mortality were IGF-1 values, the presence of severe renal dysfunction and the valvular etiology (Table 3A). Analyzing the factors associated with higher mortality in patients younger than 65 years (Table 3B), the development of fatal CV events was independently associated with the presence of the high-risk pattern and depended also on serum IGF-1 levels at the time of the enrollment.

**Table 3:** Cox regression modelling for evaluation of the associations with the outcome in the high-risk population (A) and in the young-adult population (aged < 65 years) (B)

	HR	95% C.I	*p* value
A			
High-risk patients			
Ln (IGF-1)	0.34	0.16–0.67	0.003
HF Etiology			
Idiopathic	*Ref.*		
Ischemic	2.41	1.00–5.81	0.05
Valvular	5.85	1.36–25.18	0.02
CKD-EPI stage			
I-II	*Ref.*		
III	2.03	0.91–4.51	0.08
IV-V	5.90	1.83–18.91	0.002
Systolic Blood Pressure	0.97	0.95–0.99	0.02
Heart Rate	1.00	0.96–1.03	0.72
B-Blockers	1.27	0.27–5.89	0.76
ACE-I	0.72	0.33–1.55	0.40
Diuretics	1.03	0.22–4.69	0.97
B			
Age < 65 years			
High-risk patients	10.31	1.34–79.18	0.025
Ln (IGF-1)	0.28	0.11–0.74	0.010

*HR* Hazard Ratio, *C.I.* Confidential Interval, *IGF-1* Insuline-like Growth Factor-1, *HF* Heart Failure

## Discussion

The main findings of the present analyses are that in a cohort of HFrEF the serum IGF-1 level independently predicted CV mortality, more so in younger patients and regardless of HF severity and renal function.

Prior work showed that the serum IGF-1 was a predictor of mortality from cardiovascular events in patients with HFrEF [24]. Our findings confirmed and extended the prognostic value in both the young-adult and the high-risk population (therefore, candidates for treatment with implantable cardioverter-defibrillator) [1]. The usefulness of measuring the serum levels of IGF-1 in patients with HFrEF appears prognostically crucial especially in younger subjects, regardless of the degree of renal and cardiac impairment. In such patients the presence of reduced eGFR and increased circulating BNP, usually considered as a prognostic factor in patients with HF [25–27], tend to lose their predictive role towards mortality compared with serum IGF-1.

Evaluation of IGF-1 is important because this hormone can be considered as a modifiable factor. Previous studies showed that exogenous administration of GH in patients with HF induces an improvement in functional and instrumental performances. Indeed, the study conducted by Cittadini et al. on a cohort of 17 patients treated with GH (compared with 14 control patients) [28] showed not only improved instrumental performances based on echocardiographic measurements, but also functional improvements in relation to the clinical conditions of patients, after a follow-up of 4 years. Patients were evaluated through the administration of the “Minnesota Living With Heart Failure Questionnaire” [29] (− 9.81 vs. 1.40 points, *p *= 0.001) and the changes in the NYHA class (− 0.38 ± 0.14 vs. 0.42 ± 0.17, *p *= 0.001). Beneficial effects of exogenous administration of GH were also highlighted in the case of shorter treatments (up to 6 months) in a cohort of 28 patients enrolled in the T.O.S.CA. registry [8]. In this study, positive effects due to hormonal treatment were both functional (gain in EF values) and clinical (lower duration of physical exercise).

Similar effects on EF were also shown in an Iranian study for shorter period of GH administration (up to 3 months) in patients with post-myocardial infarction HF and reduced EF (8 treated patients compared with 8 controls). However, the beneficial effect tended to decrease after about 9 months from the interruption of the therapy [30].

This indirectly indicates the clinical and functional importance of IGF-1 in patients with HF and the beneficial and protective effects by this hormone towards physical performances in a highly prevalent disease such as HF itself.

At the same time, it is also important to underline how a meta-analysis conducted in 2007 showed how the beneficial effects of GH administration in amelioration of EF (+ 5.1%), diameter of the left ventricle (− 2.02 mm), NYHA class (− 0.97) and duration of physical exercise (+ 103.7 s) were influenced by sex, being more favorable in males than females [31]. The causes of such sex-dependent differences can be partially explained by the different hormonal asset of males and females, but further studies are still needed for the identification of the leading factors.

Cittadini et al. [8] demonstrated that exogenous administration of GH led to a significant reduction in circulating N-terminal pro-BNP levels. As highlighted by our study, the serum values of IGF-1 seem to correlate only with serum BNP, which represents one of the main prognostic indicators clinically used for the evaluation of patients with HF. The association between serum BNP and worse outcomes was not found when we performed the multivariate analyses, suggesting the greater statistical strength of circulating IGF-1.

Our results did not show any relationship between serum IGF-1 and EF or with the degree of renal impairment, confirming that serum IGF-1 would not seem to be influenced by the presence of such abnormalities. These findings are in agreement with the study of Ravassa et al. on a group of 686 patients with HF. These authors showed no relationship between IGF-1 and renal dysfunction, while a significant difference was found in circulating protein binding insulin-like growth factor 2 (IGFBP-2), whose levels are usually higher in patients with impaired renal function [32]. It is known that IGF-1 functionality is affected by IGFBP-2 levels (98% of IGF-1 is bound to this protein) [33], so that an increase in IGFBP-2 levels in patients with CKD affects the peripheral function of the hormone and seems to be partially responsible for the association with a higher risk of mortality in patients with HF and CKD. Also, IGFBP-2 is an important prognostic factor for cardiovascular mortality in HF and it might be a novel therapeutic target in the treatment of HF [34, 35].

Neprilysin inhibitors have been recently introduced in the management of patients with HF and concomitant renal insufficiency levels [36], with a clinical net benefit for CV and renal outcomes, especially among young adults and in patients with HFrEF [37]. Neprilysin inhibitors seem to act in part on the GH/IGF-1 axis, as they reduce the circulating levels of insulin-like growth factor-binding protein-7 (IGFBP-7), which is a modulator of the biological activities of IGF-1 [38]. In normal conditions, IGFBP-7 binds to IGF-1 and neutralizes its activity, similarly to what happens with IGFBP-2. By acting on the IGFBP-7/IGF-1 ratio, in fact, neprilysin inhibitors can effectively modify the prognosis of patients with HF by modulating the final effector represented by IGF-1 too.

This study has several limitations: first of all its retrospective nature. Moreover, our analyses did not take into account patients’ comorbidities that could increase mortality in subjects with HF. It has to be considered that comorbidities, such as diabetes mellitus and CKD have a negative prognostic effect on patients with HF,

consequent to alterations in the serum levels of IGF-1. Furthermore, none of the patients were under sacubitril/valsartan therapy. This was because this treatment was not recommended by current guidelines at the time of enrollment. Moreover, this drug cost is refundable in Italy only starting from 2018.

Serum levels of IGF-1 may also be affected by pulsatile GH secretion. In healthy patients, GH is secreted according to a complex circadian rhythm and with different secretory peaks between males and females in terms of both the time of onset and the duration of the secretory peaks themselves [39, 40]. For these reasons, the time of execution of sampling would have been inevitably affected by such biological fluctuations in the hormonal secretion.

In addition, some drugs normally used in clinical practice, such as ACE inhibitors, could induce a modification in the serum concentrations of IGF-1 and its binding proteins, with a more significant effect in patients with HFrEF [ 15, 41 ]. This may represent a further potential explanation to the beneficial effects of ACE-inhibition in patients with HF. The role of proton pump inhibitors (PPIs) also deserves some attention, as such drugs are being widely used. PPIs seem to have a marginal part in reducing serum IGF-1 [42]. Another limitation on the interpretation of our data is that different level of physical activity might influence IGF-1 serum levels. Unfortunately, the extent of daily physical activity has not been collected in our study.

Among the conditions that could modify the serum concentrations of IGF-1, we could also mention the association between cardiac decompensation and hepatocellular dysfunction, having cirrhosis a negative effect on IGF-1 peripheral secretion [43, 44]. Finally, none of the enrolled patients received sacubitril/valsartan as disease modifying therapy.

## Conclusions

Our study demonstrates both the clinical and prognostic utility of the IGF-1 assay in patients with HFrEF. In particular, in patients at higher cardiovascular risk, the evaluation of the GH/IGF-1 axis could be useful in predicting the prognosis and planning the therapeutic strategies. This kind of approach could represent useful clinical stratification of prognosis in patients with HF.

We believe that patients with HFrEF, would benefit from early detection of GH/IGF-1 alterations, in order to adequately predict their prognosis. At the same time, clinicians could eventually plan correction of hormonal alterations, with the aim of improving long-term outcomes.

## Supplementary Information

Below is the link to the electronic supplementary material.

Supplementary file1 (DOCX 128 KB)

## Data Availability

Data supporting reported results could be obtained from authors after reasonable request.
