# Supplementary figures and images for: Insulin-like growth factor-1 (IGF-1) as predictor of cardiovascular mortality in heart failure patients: data from the T.O.S.CA. registry
Source: Intern Emerg Med. 2022 Apr 21;17(6):1651–60. doi: 10.1007/s11739-022-02980-4 (PMC9463276; doi:10.1007/s11739-022-02980-4)

Supplemental material

Kaplan Meier Curve divided by IGF-1 strata


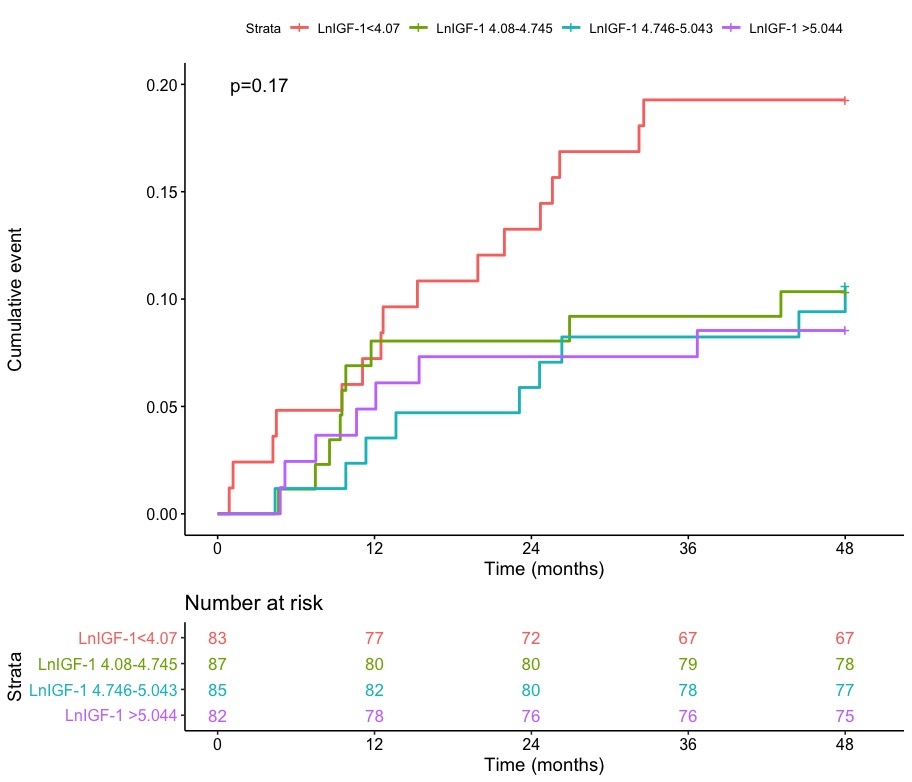


AUC plot


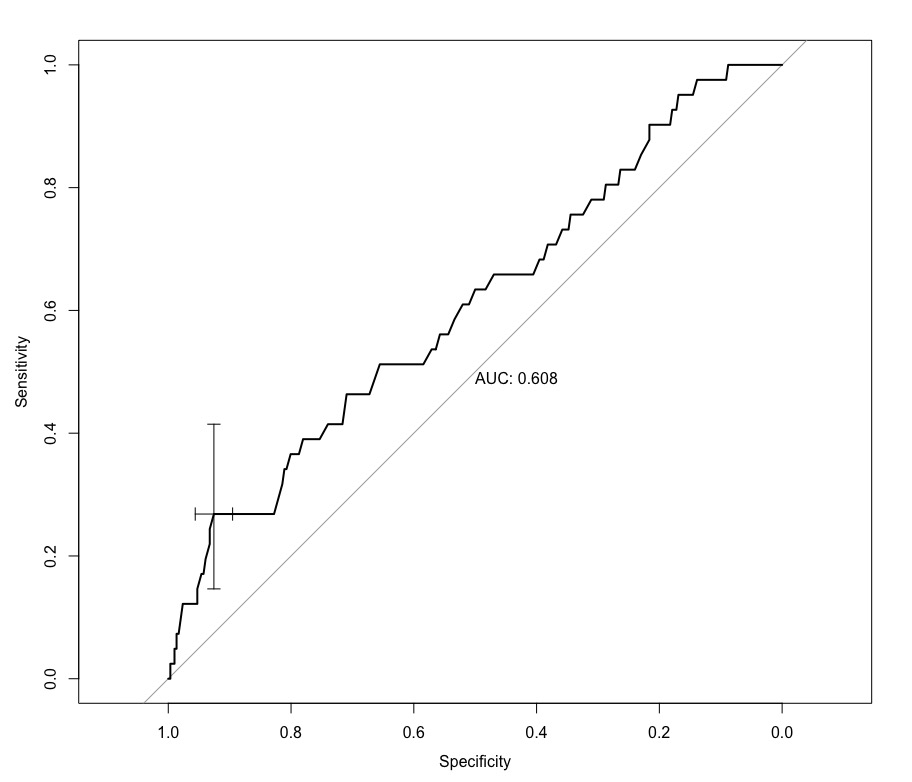

Supplement: Supplementary file 1 — Supplementary file1 (DOCX 128 KB) [file 11739_2022_2980_MOESM1_ESM.docx]
